# Antibiotic Utilization Patterns in Patients with Ventilator-Associated Pneumonia: A Canadian Context

**DOI:** 10.1155/2016/3702625

**Published:** 2016-07-20

**Authors:** Tracy Chin, Barry Kushner, Deonne Dersch-Mills, Danny J. Zuege

**Affiliations:** ^1^Department of Pharmacy Services, Alberta Health Services-Calgary Zone, Calgary, AB, Canada; ^2^Critical Care, Department of Pharmacy Services, Alberta Health Services-Calgary Zone, Calgary, AB, Canada; ^3^Pediatrics, Department of Pharmacy Services, Alberta Health Services-Calgary Zone, Calgary, AB, Canada; ^4^Departments of Critical Care Medicine and Medicine, Cumming School of Medicine, University of Calgary, Peter Lougheed Centre, Alberta Health Services-Calgary Zone, Calgary, AB, Canada

## Abstract

This retrospective cohort study describes the patterns of antibiotic use for the treatment of ventilator-associated pneumonia (VAP) in the Calgary Zone of Alberta Health Services. Timing, appropriateness, and duration of antibiotics were evaluated in two hundred consecutive cases of VAP derived from 4 adult intensive care units (ICU). Antibiotic therapy was initiated in less than 24 hours from VAP diagnosis in 83% of cases. Although most patients (89%) received empiric therapy that demonstrated* in vitro* sensitivity to the identified pathogens, only 24% of cases were congruent with the 2008 Association of Medical Microbiology and Infectious Disease (AMMI) guidelines. Both ICU (*p* = 0.001) and hospital (*p* = 0.015) mortality were significantly lower and there was a trend for shorter ICU length of stay (*p* = 0.051) in patients who received appropriate versus inappropriate initial antibiotics. There were no outcome differences related to compliance with AMMI guidelines. This exploratory study provides insight into the use of antimicrobials for the treatment of VAP in a large Canadian health region. The discordance between the assessments of appropriateness of empiric therapy based on recovered pathogens versus AMMI guidelines is notable, emphasizing the importance of using as much as possible local microbiologic and antimicrobial resistance data.

## 1. Introduction

Ventilator-associated pneumonia (VAP) occurs in 10–20% of intubated patients and is one of the most frequent nosocomial infections occurring in intensive care patients [1]. VAP is associated with increased duration of mechanical ventilation [1–4], length of hospital stay [1–4], and hospital costs [1–3]. In the Alberta Health Services-Calgary Zone (AHS-Calgary Zone), each VAP case is estimated to increase hospital costs by about $30,000 [5].

Optimal antibiotic therapy is the major focus of VAP treatment. Factors associated with antibiotic therapy, which previous studies have identified as influencing patient outcomes, include the rapidity of initiation of antimicrobial therapy after a diagnosis of VAP is made, the duration of treatment, the adequacy of initial antibiotics relative to identified pathogens, and changes to antimicrobial therapy in response to culture and sensitivity results [4]. On the basis of these observations, a number of clinical practice guidelines for antibiotic therapy for VAP have evolved over the years [4–7]. Virtually, all of these guidelines are based on studies from diverse patient populations, including different countries, types of intensive care units, variations in patterns of antimicrobial use, and local incidence of complex and/or resistant pathogens. Geographically, most studies of antimicrobial use for VAP are derived from the United States and Europe with a paucity of information deriving from Canada. It is not clear whether or not existing pangeographic VAP treatment guidelines may reflect microbiologic and antibiotic resistance patterns in Canada or in AHS-Calgary Zone specifically.

In 2004, AHS-Calgary Zone implemented a number of strategies targeting the prevention of VAP and observed a concordant decrease in the incidence of VAP from 19 cases per 1000 ventilator-days in 2002 to less than 9.8 cases per 1000 ventilator-days in 2007 [5]. These strategies were focused on VAP prevention and did not address optimization of VAP treatment, which has not previously been formally assessed in our region [5]. The primary goal of this study was to describe the patterns of antibiotic use for VAP treatment in AHS-Calgary Zone. The information from this project will be used to identify targets for quality improvement in antimicrobial utilization for VAP treatment within our region. In addition, this data will enhance the understanding of differences in VAP microbiology and antibiotic resistance patterns that may exist between Canada and other areas of the world.

## 2. Methods

This retrospective cohort study included all patients diagnosed with VAP from April 1, 2007, to May 31, 2010, in the four adult multidisciplinary intensive care units (ICU) in AHS-Calgary Zone which care for all critically ill patients within the city of Calgary and surrounding regions. This time period was chosen to be large enough to include a reasonable number of cases of VAP yet contemporary enough to reflect current antibiotic utilization patterns.

The primary objectives of this study were to describe the following in patients with VAP:

- The timing of empiric antibiotic therapy.
- The appropriateness of empiric antibiotic therapy based on the cultured pathogens.
- The appropriateness of empiric antibiotic therapy based on criteria presented in the 2008 Association of Medical Microbiology and Infectious Disease (AMMI) guidelines (Appendix B) [4].
- The duration of antibiotic therapy.

Secondary objectives were to describe the relation between measures of antibiotic utilization (timing, appropriateness, and duration of therapy) and patient outcomes (ICU length of stay, duration of mechanical ventilation, and ICU and hospital mortality).

### 2.1. Identification of VAP Cases

Patients with VAP were identified via a well-established zonal VAP surveillance program, described in detail elsewhere [5], which operated continuously during the study period. Case finding was accomplished via the identification of triggers occurring during the ICU stay, most commonly microbiological (performance of any respiratory, pleural, or blood cultures) though also radiological (direct review or chest X-ray report suggesting nosocomial pneumonia) or clinical (suspicion of VAP based on discussion with healthcare professionals or record in the patient's chart). The identification of any trigger initiated a formal case review in order to identify cases of VAP. Case reviews were performed by a dedicated group of critical care infection control practitioners, who were not directly involved in the patient's care, using a VAP surveillance database populated with demographic, clinical, radiologic, microbiologic, and antibiotic data. Inconclusive cases were subject to group review which included all infection control practitioners and a critical care physician. The case definition used in the surveillance system (Appendix A) is based on the Centers for Disease Control and Prevention definitions for VAP in place at the time [8] and remained constant for the duration of this study.

### 2.2. Study Definitions

The date and time of VAP were defined as the date and time of the first chest X-ray supporting the diagnosis of VAP. Growth of a pathogen in any culture of blood, pleural fluid, lung tissue, bronchoalveolar lavage, bronchoscopic protected brush, or endotracheal aspirate in a patient with VAP was considered to be a positive VAP culture [9]. If multiple positive cultures were identified, only pathogens identified in cultures drawn within 48 hours of a diagnosis of VAP were counted [9]. Cases with positive microbiology were divided into two groups: Group I, where cultures did not include growth of any species of* Acinetobacter, Stenotrophomonas,* or* Pseudomonas*, and Group II, where cultures did include significant growth of one or more of* Acinetobacter, Stenotrophomonas,* or* Pseudomonas* species.

### 2.3. Study Analysis

**Figure 1:**
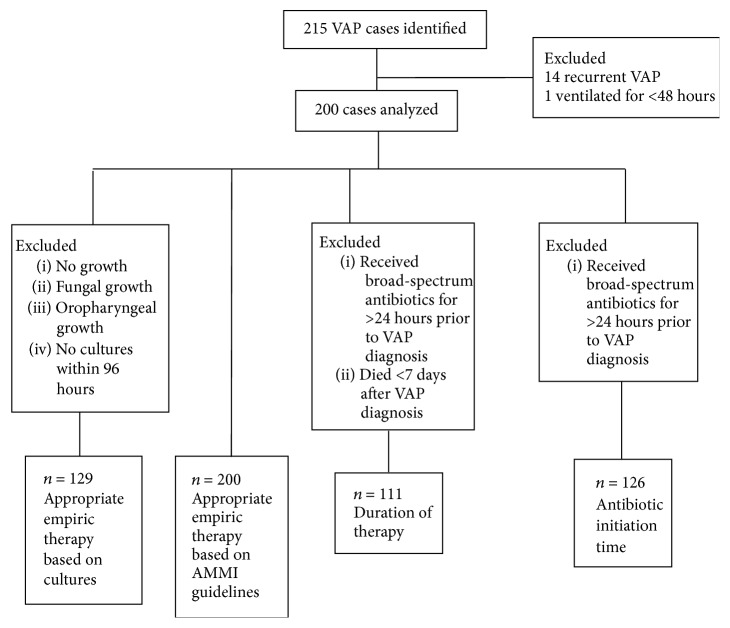
Study participants and analysis schema.

The analysis for each measure had distinctive predefined sets of exclusion and inclusion criteria (shown in Figure 1). For all analyses, when patients experienced multiple episodes of VAP during the same hospitalization, only the first episode was included.

### 2.4. Antibiotic Timing Analysis

The timing of antibiotic initiation was defined as the time from VAP diagnosis to the first dose of a new antimicrobial. Patients treated with broad-spectrum antibiotics for greater than 24 hours prior to VAP were excluded from the analysis of antibiotic timing.

### 2.5. Appropriate Empiric Therapy Analysis

Two definitions of appropriate empiric therapy were applied.

*(a) Culture-Focused Definition*. Empiric therapy was considered appropriate when the isolated pathogens associated with VAP were sensitive to at least one antimicrobial agent initiated prior to culture results becoming available [10, 11]. In evaluating appropriate empiric therapy, if the patient was receiving antibiotics prior to the diagnosis of VAP and these antibiotics were continued after VAP diagnosis, these were considered as the intended VAP therapy. Organisms reported as being “susceptible” were considered to be sensitive. Pathogens reported as “intermediate” and “resistant” were considered to be not sensitive [12]. Therapy was considered “inappropriate” if one or more pathogens were not sensitive to any of the antibiotics currently being administered [12]. For antibiotics that were not listed on the sensitivity report, the Calgary Zone regional antibiogram reflective of the most current susceptibility profiles at the time of the patient's ICU admission was used to determine appropriateness, if available. For antibiotic therapy not reported in the sensitivity report or the regional antibiogram, Sanford's Guide to Antibiotic Therapy was used to evaluate appropriateness (this was used in less than 5% of the cases to determine appropriate therapy) [13]. If no cultures were drawn within 96 hours of VAP diagnosis or if the final microbiology report showed any of the “no growth,” “fungal growth,” or growth of oropharyngeal flora, the VAP case was excluded from the analysis of appropriate empiric therapy based on culture and sensitivity reports.

*(b) Literature-Focused Definition*. Antimicrobial treatment was considered appropriate if the therapies initiated prior to reporting of culture results were consistent with the 2008 AMMI guidelines (Appendix B) [4]. AMMI guidelines list vancomycin as an optional therapy dependent on the presence or suspicion of methicillin-resistant* Staphylococcus aureus* (MRSA). Vancomycin use in the current study followed AMMI guidelines and was regarded as optional therapy. If the patient was receiving antibiotics prior to the diagnosis of VAP and these antibiotics were continued after VAP diagnosis, these were considered as the intended VAP therapy.

### 2.6. Antibiotic Duration Analysis

Appropriate duration of therapy for Group I bacteria was defined as antimicrobial treatment for 7 to 9 days [4, 13] and for Group II bacteria for 13 to 15 days [4]. VAP cases receiving broad-spectrum antibiotics greater than 24 hours prior to VAP diagnosis or who died within 7 days of VAP diagnosis were excluded from the duration of therapy analysis. Fifteen cases were excluded on the basis of death within 7 days of diagnosis, ten (66%) of these cases receiving 3 days or less of antibiotics.

### 2.7. Data Sources and Statistics

Data for this study was collected from a local critical care database (TRACER, Microsoft Oracle) via a structured query. Information requiring clarification was verified from the local patient care information system (Sunrise Clinical Manager, Allscripts). Collected data included patient demographics (age, sex, allergies, ICU admission class (medical, surgical, trauma, and neurological), and admission Acute Physiology and Chronic Health Evaluation (APACHE) II score); ICU admission data (date and times of ICU admission and use of invasive mechanical ventilation); infection parameters (results of all microbial cultures performed during ICU admission, date and time of VAP diagnosis, and type and timing of all antibiotics administered before and after VAP diagnosis); patient outcome data (ICU and hospital survival and durations of ICU stay and mechanical ventilation). The results of Gram stains were not available for this study.

Given that this study was primarily descriptive and performed retrospectively, a power analysis was not performed. The sample size reflected the chosen analysis time frame which reflected when data was available and a reasonably contemporary time range of antibiotic utilization patterns. Descriptive analyses were performed for age, sex, APACHE II score, and ICU admission class in addition to the variables forming the primary and secondary measures. Data are reported as means and standard deviations or medians and interquartile ranges as appropriate based on analysis of normal distribution visually and statistically (Shapiro-Wilk test). ICU length of stay, duration of mechanical ventilation, and ICU and hospital mortality were compared between groups related to antibiotic appropriateness, timing, and duration of therapy using analysis of variance, Kruskal-Wallis, and chi-squared tests as appropriate. A two-sided *p* value of <0.05 was used to indicate statistical significance. Statistical analyses were performed using SPSS version 20.0 (IBM) and Microsoft Excel 2007.

This study was approved by the University of Calgary Conjoint Health Research Ethics Board, which waived the need for informed consent. No outside funding was provided for this study.

## 3. Results

Two hundred consecutive cases of VAP were identified from April 1, 2007, to May 31, 2010 (Figure 1). The number of cases analyzed for different objectives varied based on predefined analysis exclusion criteria for each measure (Figure 1). Patient characteristics are described in Table 1. VAP was diagnosed within a median of 5.8 (IQR: 3.7 to 10.7) days after ICU admission with 70% of cases constituting “late VAP” (diagnosis greater than 4 days after ICU admission).

Cultures grew oropharyngeal flora in 26% of cases and cultures were negative in 7% of cases (Table 1). There were a greater number of total pathogens relative to the number of VAP cases due to multiple pathogens being identified in a number of cases. Isolated VAP bacterial pathogens are listed in Table 2. Methicillin-sensitive* Staphylococcus aureus* (MSSA) was the most common pathogen identified.

Initial empiric antibiotic therapies directed towards VAP are listed in Table 3. Piperacillin-tazobactam was the most common empiric antibiotic used. The total number of antibiotic agents used was greater than the number of VAP cases given the use of multiple antibiotics in several cases. Cefazolin was used in 28 (14%) of VAP cases, 12 of these cases being postoperative and 10 trauma-related ICU admissions, with continuation of therapy present at ICU admission after a diagnosis of VAP.

One hundred twenty-six cases were eligible for the analysis of empiric antibiotic timing. The median time to antibiotic initiation was 3.7 (IQR: 0 to 13.2) hours. Antibiotic therapy was initiated in less than 12 and 24 hours from VAP diagnosis in 74% and 83% of the cases, respectively (Table 4). Forty cases had antimicrobial therapy initiated before the assigned time of VAP: in most cases this likely related to initiation of therapy prior to the performance of chest imaging based on the presence of other criteria for sepsis.

One hundred twenty-nine patients were eligible for analysis of appropriate empiric therapy based on cultures. All patients (*n* = 200) were eligible for analysis of appropriate empiric therapy based on AMMI guidelines (Figure 1). Eighty-nine percent of patients received appropriate empiric therapy that demonstrated* in vitro* sensitivity to the identified pathogens (Table 4). Of the 14 cases that received inappropriate empiric therapy, five cases grew Group II bacteria (*Stenotrophomonas* and* Pseudomonas* species), three cases grew methicillin-sensitive* Staphylococcus aureus*, two cases grew extended-spectrum beta-lactamase (ESBL) producing* Klebsiella* species, two cases grew* Citrobacter freundii*, one case grew* Klebsiella pneumoniae*, one case grew* Haemophilus influenzae*, and one case grew methicillin-resistant* Staphylococcus aureus*. Three of the 14 cases did not receive antibiotic therapy. In only 24% of the cases was empiric therapy congruent with the 2008 AMMI guidelines (Table 4).

The median duration of antibiotic therapy for all VAP cases was 10 (IQR: 7 to 14) days (Table 5). The median duration of therapy for Group I cases was shorter than for Group II cases though this was not statistically significant (*p* = 0.12). Only 38% of Group I cases had durations of antibiotic therapy of 7 to 10 days (Table 5). Only 4 (50%) of Group II cases had durations of therapy within the 13-to-15-day range. Of note, the median duration of therapy for methicillin-resistant* Staphylococcus aureus* was 13 days.

For the overall cohort, the median ICU length of stay was 18.0 (IQR: 11.6 to 27.6) days and the median duration of ventilation was 16.5 (IQR: 11.0 to 25.0) days (Table 4). ICU and hospital mortality were 23% and 32%, respectively (Table 4).

**Figure 2:**
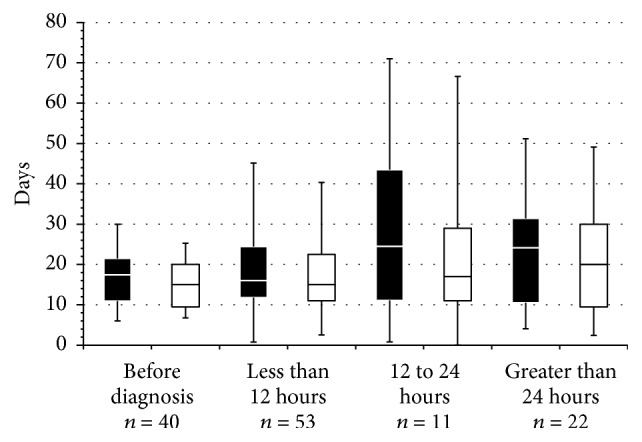
ICU length of stay and duration of mechanical ventilation related to antibiotic initiation time. Box plots of ICU length of stay (■) and duration of mechanical ventilation duration (□) related to antibiotic initiation time. Box plots show the median value and 25th and 75th percentiles; the whiskers show the mean ± 1 standard deviation. Numerical differences were not statistically significant.

**Figure 3:**
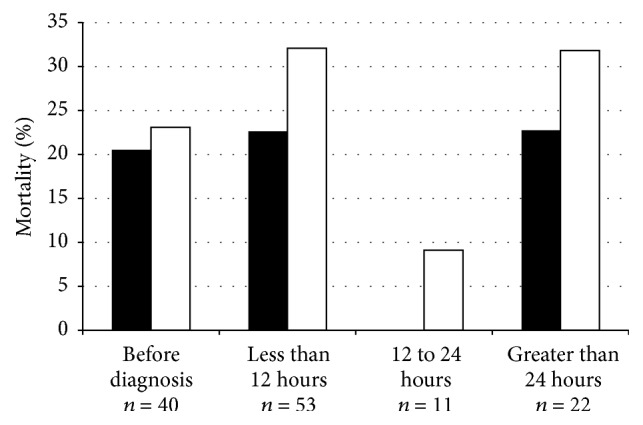
ICU and hospital mortality related to antibiotic initiation time. There was no significant difference in ICU mortality (■; *p* = 0.37) and in hospital mortality (□; *p* = 0.47) between the four antibiotic initiation time groups. Data presented as percentages.

Data relating the timing of initial antibiotics and ICU length of stay, duration of mechanical ventilation, and mortality are shown in Figures 2 and 3. Earlier empiric antimicrobial initiation was associated with shorter ICU length of stay and duration of mechanical ventilation, neither of which was statistically significant (Kruskal-Wallis tests; *p* = 0.22 and 0.32, resp.) (Figure 2). ICU and hospital mortality ranged from 0 to 23% and from 9 to 32%, respectively, among the different groups related to antibiotic initiation time with no statistical difference in ICU (Fisher's Exact test = 3.19; *p* = 0.37) or hospital (Fisher's Exact test = 2.60; *p* = 0.47) mortality between groups (Figure 3).

**Figure 4:**
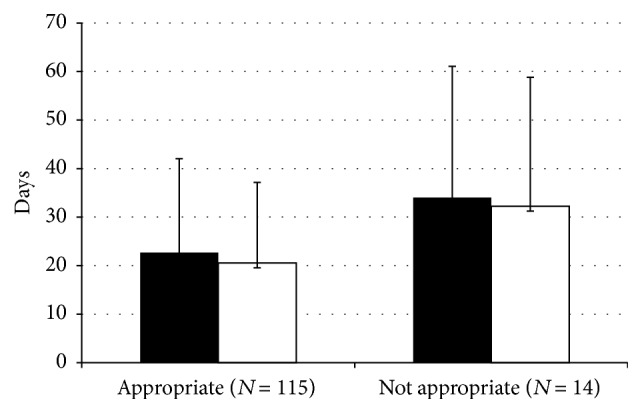
ICU length of stay and duration of mechanical ventilation related to antibiotic appropriateness based on culture results. Overall trend for shorter ICU length of stay (■; *p* = 0.051) for those that received appropriate empiric antibiotic therapy when compared to the group that received inappropriate empiric antibiotics. Duration of mechanical ventilation (□; *p* = 0.13) was numerically but not significantly shorter in those who received appropriate empiric antibiotics. Data presented as mean ± standard deviation.

**Figure 5:**
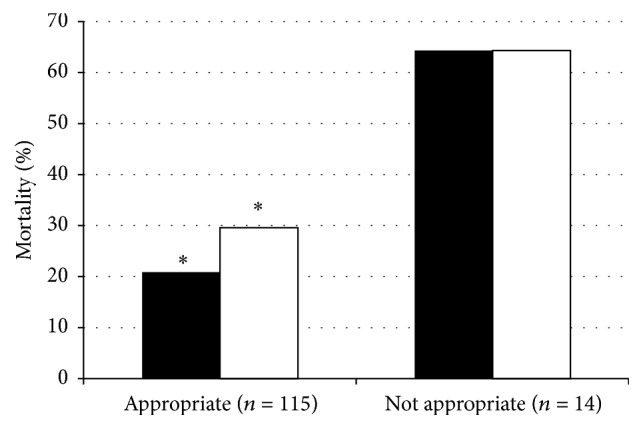
ICU and hospital mortality related to antibiotic appropriateness based on culture results. Both ICU (■) and hospital (□) mortality were significantly less in the group that received initially appropriate empiric antibiotics for VAP (^*∗*^ *p* < 0.02 for both). Data presented as percentages.

ICU length of stay was numerically and near statistically shorter for the VAP cases that were sensitive to empiric antimicrobial therapy (22.7 ± 19.4 days) when compared to VAP cases that were insensitive to empiric therapy (34.0 ± 27.1 days) (difference: 11.3 days (95% CI: 0.07–22.7); *p* = 0.051) (Figure 4). Mechanical ventilation duration was also numerically but not significantly shorter in the group that received culture-sensitive empiric antibiotics (20.6 ± 16.6 days) when compared to the group that received inappropriate empiric antibiotics based on cultures (32.2 ± 26.6 days) (difference: 11.7 days (95% CI: 1.7–21.8); *p* = 0.13) (Figure 4). Both ICU (*X* ^2^ = 12.4, *p* = 0.001) and hospital (*X* ^2^ = 6.8, *p* = 0.015) mortality were significantly lower in the group of cases where the VAP pathogens were sensitive to the empiric antibiotics chosen relative to when one or more pathogens were resistant to empiric therapy (Figure 5). There were no differences in ICU length of stay, duration of mechanical ventilation, or ICU and hospital mortality between cases compliant and not compliant with AMMI guidelines (data not shown).

**Figure 6:**
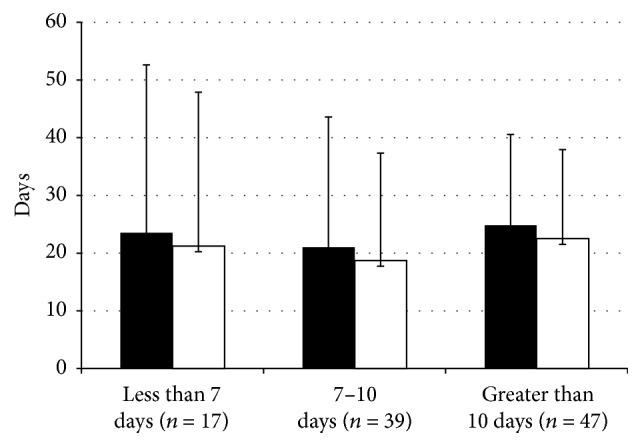
ICU length of stay and duration of mechanical ventilation related to length of antibiotic therapy in Group I bacteria. ICU length of stay (■) and duration of mechanical ventilation duration (□) in patients treated with less than 7 days, 7 to 10 days, and greater than 10 days of antibiotic therapy for Group I bacteria. There were no significant differences in ICU length of stay (*p* = 0.71) or duration of mechanical ventilation (*p* = 0.65) among the duration groups. Data presented as mean ± standard deviation.

Data relating the duration of antibiotic therapy to outcome is shown in Figure 6. Provision of 7–10 days of therapy in Group I was associated with numerically shorter durations of ICU stay and mechanical ventilation, though neither was statistically significant (ANOVA *F*(2,100) = 0.35, *p* = 0.71; ANOVA *F*(2,100) = 0.37, *p* = 0.65, resp.). Group II cases did not demonstrate any associations between duration of therapy and outcomes. ICU and hospital mortality did not differ in Group I patients between strata of antibiotic durations.

## 4. Discussion

Appropriate utilization of antimicrobial therapy relative to the timing of initiation of empiric therapy, appropriateness of initial antibiotics chosen, and duration of therapy is recurrently identified as influencing the outcome from VAP [4, 6, 14–19]. Numerous clinical practice guidelines have evolved over the past years reflecting these findings. The majority of these guidelines, however, do not reflect local microbiologic data or antimicrobial resistance patterns, limiting their generalizability to a given unit, hospital, region, or country. There is a paucity of Canadian studies investigating antibiotic utilization in VAP. Our study evaluates antimicrobial therapy for VAP within a Canadian healthcare context relating antibiotic use to local microbiologic data in a sizeable cohort of VAP cases within a large healthcare region. Our study identified the notion that although antimicrobial use was largely compliant with current recommendations for timing of therapy and the empiric therapies provided were appropriate to the pathogens identified in most cases, there remained room for improvement in each of these aspects.

Late-onset VAP is generally associated with a higher likelihood of multidrug resistant bacteria and worse patient outcomes relative to early-onset VAP [2, 4, 20]. The pathogens identified in our study more closely resembled those generally associated with early-onset VAP, despite the majority (70%) of cases in our cohort being diagnosed greater than 4 days after ICU admission [2, 4, 6]. These findings are likely explained by differences in local microbial ecology and antimicrobial use between centers highlighting the importance of incorporating local microbiologic and antibiotic resistance data when treating individual cases of VAP and when assessing the quality of care for VAP. Of note, the culture incidence pattern identified in our study is similar to that reported in a large Canadian diagnostic study for VAP [21].

Previous studies have highlighted the importance of early appropriate antibiotic administration in the survival of critically ill patients with infection [22]. Receipt of appropriate antibiotics greater than 24 hours after onset of VAP was identified as an independent risk factor for hospital mortality [23]. Our findings show that 17.5% of patients commenced appropriate empiric therapy greater than 24 hours after diagnosis, which remains suboptimal despite being somewhat better than what is currently reported in the literature [12]. Consistent with prior studies, our data revealed that earlier initiation of antibiotic therapy was associated with numerically shorter durations of ICU stay and mechanical ventilation. Although we did not observe a relationship between the timing of antimicrobial therapy and hospital or ICU mortality in our study, our sample size was relatively small and we did not account for additional patient prognostic factors.

Eighty-nine percent of VAP patients received appropriate empiric antimicrobial therapy relative to the pathogens cultured. This is comparable to previous studies where 78–85% of reported VAP cases received culture-appropriate empiric therapy [16, 17, 24]. The use of appropriate antimicrobials with* in vitro* activity against identified pathogens has been recurrently associated with improved survival and less acquisition of antimicrobial resistance [6, 16, 18, 25]. Our study reflects these observations with significantly higher ICU and hospital survival and a trend to shorter length of ICU stay in patients who received culture-sensitive empiric therapy.

In our study, only 24% of VAP cases were given empiric therapy congruent with the 2008 AMMI guidelines. The largest source of noncongruence, found in 58% of noncongruent cases, was failure to initiate two antipseudomonal agents in cases where this is indicated by AMMI guidelines (Appendix B). Other studies have also identified noncongruence with existing practice guidelines for antimicrobial therapy for nosocomial pneumonia, the most commonly reported reason for noncompliance similarly being failure to use secondary Gram negative agents [24, 26]. Although many practice guidelines suggest using a combination of two or more antibiotics to reduce risks of neglecting potentially resistant pathogens in at-risk patients [4, 6], these guidelines do not generally reflect the heterogeneity of local culture and susceptibility patterns. Our study disclosed no outcome differences between patients provided with empiric antibiotic therapy compliant with AMMI guidelines* versus* those noncompliant with guidelines. This is consistent with a recent retrospective study comparing VAP patient outcomes relative to compliance with American and Japanese VAP treatment guidelines [26]. Such data suggests limitations to the role of generalized guidelines in solely directing local therapy for VAP.

Inadequate duration of antibiotic therapy may not sufficiently treat an infection and may result in relapse; however, antibiotics of extended duration may increase chances of antibiotic toxicity, acquisition of multidrug resistant bacterial and/or fungal infections, and higher drug expenditures [4, 19, 27]. Chastre et al. demonstrated that 8 (*versus* 15) days of antibiotic therapy for VAP did not result in adverse outcomes in terms of mortality, length of ICU stay, and rate of recurrent infections [19], findings corroborated by others [11, 16]. Our study found no significant outcome differences for patients with Group I pathogens over strata of antibiotic durations. Associations were harder to assess for Group II pathogens because of the limited sample size (*n* = 8).

Strengths of this study include a relatively large sample of consecutive patients with VAP from a health region comprised of multiple acute care sites which provide the totality of critical care to adults; the use of a well-established VAP surveillance system with consistent validated definitions, processes, and personnel throughout the study duration; and the use of robust electronic sources for microbiology and antimicrobial data which capture all instances of this data in our health region. Our study also has limitations. This includes a limited ability to identify concurrent infections and non-VAP infections predating VAP diagnosis. To minimize the limitation of this confounder, those who were started on broad-spectrum antibiotics greater than 24 hours before a diagnosis of VAP were not included in the analyses for timing of initial therapy and duration of therapy. We defined use of antimicrobials of intermediate sensitivity to the identified pathogens as inappropriate therapy. Though in clinical practice pathogens may be successfully treated with antimicrobials with intermediate susceptibility, there remains significant opportunity for therapeutic failure in this circumstance. In order to improve the generalizability of our results for this study, we defined appropriate antimicrobials as agents reported as fully sensitive against the relevant pathogens. Our analyses of patient outcomes were not adjusted for other predictors of mortality. Finally, given the data for this study derived solely from the Calgary Zone, the results may not be generalizable to other centers where microbiological patterns may differ.

This exploratory study provides insight into the use of antimicrobials for the treatment of VAP patients in relation to timing, duration, and appropriateness of empiric therapies in a large Canadian health region. The discordance between the assessments of appropriateness of empiric therapy based on recovered pathogens versus AMMI guidelines is notable emphasizing the importance of using local microbiologic and antimicrobial resistance data in formulating optimal treatment regimens prospectively and for the assessment of the adequacy of treatments applied for quality improvement purposes retrospectively. Our study adds to the limited amount of VAP-related antimicrobial utilization data deriving from Canada and contributes to a Canadian context to guidelines for the treatment of VAP.

## Figures and Tables

**Figure 7:**
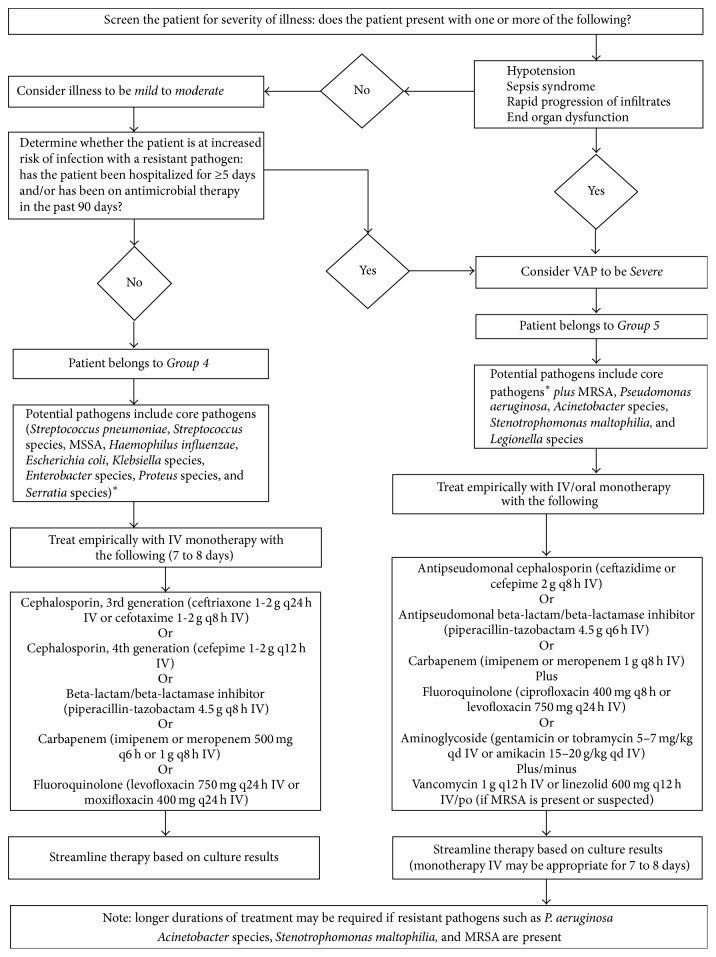
^*∗*^Core pathogens include* Streptococcus pneumoniae*,* Streptococcus* species,* Haemophilus influenzae*,* Enterobacter* species,* Escherichia coli*,* Klebsiella* species,* Proteus* species,* Serratia marcescens*, and methicillin-susceptible* Staphylococcus aureus*. 2008 VAP Treatment Algorithm. This figure is republished with permission.

**Table 1:** Patient characteristics (total number of VAP cases = 200).

Age, years, median (interquartile range)	47.9 (26.3–61.3)
Male sex	149 (74.5)
APACHE II score, median (interquartile range)^*∗*†^	19 (15–24)
<24	147 (74.2)
≥24	51 (25.8)
Admission class	
Medical	42 (21.0)
Surgical	71 (35.5)
Neurological	24 (12.0)
Trauma	63 (31.5)
Time from ICU admission to VAP diagnosis, days, median (interquartile range)	5.8 (3.7–10.7)
Timing of VAP, days after ICU admission	
Early (≤4 days)	61 (30.5)
Late (>4 days)	139 (69.5)
Microbiology	
No growth	13 (7)
Oropharyngeal flora	51 (26)
Yeast	5 (3)
Bacterial	131 (65)
Monomicrobial	108 (82)
Polymicrobial	23 (18)

^*∗*^Two patients were missing APACHE II data.

^†^APACHE: Acute Physiology and Chronic Health Evaluation score which ranges from 0 to 71, higher values indicating greater acuity of illness.

**Table 2:** VAP bacterial pathogens (total number of VAP cases = 200).

Bacteria	Number of pathogens (%)
*Staphylococcus aureus*	54 (27)
Methicillin-sensitive	44
Methicillin-resistant	10

*Hemophilus influenzae*	26 (13)

*Enterobacter cloacae*	14 (7)

*Pseudomonas aeruginosa*	11 (6)

*Klebsiella *species	10 (5)
Non-ESBL	8
ESBL^*∗*^	2

*Escherichia coli*	9 (5)
Non-ESBL	8
ESBL^*∗*^	1

*Streptococcus pneumoniae*	8 (4)

*Stenotrophomonas maltophilia*	5 (3)

*Acinetobacter baumannii*	3 (2)

*Serratia marcescens*	3 (2)

*Citrobacter koseri, Citrobacter freundii *	2 (1)

Group A streptococci	2 (1)

Others^*∗∗*^	9 (5)

^*∗*^ESBL: extended-spectrum beta-lactamase producing organisms.

^*∗∗*^Including *Streptococcus milleri*, *Group B Streptococcus, Group C Streptococcus, Oligella ureolytica, Aeromonas hydrophila, Neisseria meningitidis, Moraxella catarrhalis*, and *Achromobacter xylosoxidans*, each of which was present in 1 VAP case.

Percentages add up to more than 100% given that more than one pathogen was recovered in several cases.

**Table 3:** Empiric antibiotics initiated (total number of VAP cases = 200).

Agents	Number of VAP cases (%)
Piperacillin-tazobactam	81 (41)
Vancomycin	75 (38)
Ciprofloxacin	50 (25)
Metronidazole	37 (19)
Cefazolin	28 (14)
Ceftriaxone	16 (8)
Meropenem	16 (8)
Cefuroxime	7 (4)
Gentamicin	7 (4)
Ceftazidime	6 (3)
Levofloxacin	5 (3)
Ampicillin	4 (2)
Cefotaxime	4 (2)
Cloxacillin	3 (2)
Trimethoprim-sulfamethoxazole	2 (1)
Others^*∗*^	5 (3)

*∗* includes: azithromycin, cefepime, erythromycin, linezolid and penicillin, each of which were used in 1 VAP case.

Percentages add up to more than 100% given more than one antibiotic was initiated in many cases.

**Table 4:** Antibiotic utilization and patient outcomes.

Timing of antibiotic initiation (*n* = 126)	
<12 h	93 (73.8)
12 h–24 h	11 (8.7)
>24 h	22 (17.5)
Appropriate empirical antibiotic therapy	
Based on culture results^*∗*^ (*n* = 129)	115 (89.1)
Based on AMMI guidelines (*n* = 200)	48 (24)
Mortality (*n* = 200)	
ICU	46 (23)
Hospital	63 (31.5)
ICU length of stay, days, median (IQR)	18.0 (11.6, 27.6)
Duration of mechanical ventilation, days, median (IQR)	16.5 (11.0, 25.0)

Data presented as *n* (%) unless otherwise indicated; ^*∗*^cultured pathogens were sensitive to the antibiotic therapy chosen. AMMI: Association of Medical Microbiology and Infectious Disease; ICU intensive care unit; and IQR: interquartile range.

**Table 5:** Duration of therapy according to microbiology.

	All cases (*n* = 111)^*∗*^	Group I (*n* = 103)^*∗*^	Group II (*n* = 8)^*∗*^
Duration of antibiotics: days, median (IQR)	10.0 (7, 14)	10.0 (7, 15)	13.0 (11, 13.5)

Difference		3.0 (*p* = 0.12)	

Proportion of cases with antibiotic durations of (*N* (%))			

<7 days		17 (16.5)	
7–10 days		39 (37.9)	
>10 days		47 (45.6)	

<13 days			3 (37.5)
13–15 days			4 (50)
>15 days			1 (12.5)

^*∗*^Out of 200 VAP cases, 111 grew bacterial pathogens that were further divided into Group I and Group II bacteria.

Group I: pathogens that did not include any of the *Acinetobacter, Stenotrophomonas*, or *Pseudomonas* species.

Group II: pathogens that included any of the *Acinetobacter, Stenotrophomonas*, or *Pseudomonas* species.

## Appendix

### A. VAP Case Definition [8, 9]

VAP was established when all of the following criteria were fulfilled:

1. Receiving invasive mechanical ventilation continuously (>18 hours/day) via an endotracheal tube or tracheostomy for at least 48 hours; noninvasive ventilation (Continuous Positive Airway Pressure or Bilevel Positive Airway Pressure) was not included.
2. Onset of VAP while receiving or within 48 hours of having received invasive mechanical ventilation.
3. Chest X-ray showing new or progressive infiltrates.
4. At least 1 of the following: fever (>38.0°C) or hypothermia (<35°C); altered white blood cell count (>12,000/mm^3^ or <4000/mm^3^); change in sputum: new onset purulent sputum or change in sputum character or increase in sputum volume/suctioning requirements; there should be repeated notations of altered sputum volume and/or character over 24 hours; purulence is subjective but Gram stain with >25 neutrophils and <10 cells/low power field is supportive.

### B. AMMI VAP Guidelines [4]

See Figure 7.

## References

[1] Rewa O., Muscedere J. (2011). Ventilator-associated pneumonia: update on etiology, prevention, and management. *Current Infectious Disease Reports*.

[2] Joseph N. M., Sistla S., Dutta T. K., Badhe A. S., Parija S. C. (2010). Ventilator-associated pneumonia: a review. *European Journal of Internal Medicine*.

[3] Muscedere J. G., Martin C. M., Heyland D. K. (2008). The impact of ventilator-associated pneumonia on the Canadian health care system. *Journal of Critical Care*.

[4] Rotstein C., Evans G., Born A. (2008). Clinical practice guidelines for hospital-acquired pneumonia and ventilator-associated pneumonia in adults. *Canadian Journal of Infectious Diseases and Medical Microbiology*.

[5] Esmail R., Duchscherer G., Giesbrecht J., King J., Ritchie P., Zuege D. (2008). Prevention of ventilator-associated pneumonia in the calgary health region: a Canadian success story!. *Healthcare Quarterly*.

[6] American Thoracic Society and Infectious Diseases Society of America (2005). Guidelines for the management of adults with hospital-acquired, ventilator-associated, and healthcare-associated pneumonia. *American Journal of Respiratory and Critical Care Medicine*.

[7] Muscedere J., Dodek P., Keenan S., Fowler R., Cook D., Heyland D. (2008). Comprehensive evidence-based clinical practice guidelines for ventilator-associated pneumonia: diagnosis and treatment. *Journal of Critical Care*.

[8] Horan T. C., Andrus M., Dudeck M. A. (2008). CDC/NHSN surveillance definition of health care-associated infection and criteria for specific types of infections in the acute care setting. *American Journal of Infection Control*.

[9] Infection Prevention and Control and Department of Critical Care Medicine (2010). VAP Surveillance Program. *Definitions*.

[10] Micek S. T., Heuring T. J., Hollands J. M., Shah R. A., Kollef M. H. (2006). Optimizing antibiotic treatment for ventilator-associated pneumonia. *Pharmacotherapy*.

[11] Micek S. T., Ward S., Fraser V. J., Kollef M. H. (2004). A randomized controlled trial of an antibiotic discontinuation policy for clinically suspected ventilator-associated pneumonia. *Chest*.

[12] Kollef M. H., Morrow L. E., Niederman M. S. (2006). Clinical characteristics and treatment patterns among patients with ventilator-associated pneumonia. *Chest*.

[13] Gilbert D. N., Moellering R. C., Eliopoulos G. M., Chambers H. F., Saag M. S. (2010). *The Sanford Guide to Antimicrobial Therapy*.

[14] Mathevon T., Souweine B., Traoré O., Aublet B., Caillaud D. (2002). ICU-acquired nosocomial infection: impact of delay of adequate antibiotic treatment. *Scandinavian Journal of Infectious Diseases*.

[15] Luna C. M., Aruj P., Niederman M. S. (2006). Appropriateness and delay to initiate therapy in ventilator-associated pneumonia. *European Respiratory Journal*.

[16] Leone M., Garcin F., Bouvenot J. (2007). Ventilator-associated pneumonia: breaking the vicious circle of antibiotic overuse. *Critical Care Medicine*.

[17] Kollef K. E., Schramm G. E., Wills A. R., Reichley R. M., Micek S. T., Kollef M. H. (2008). Predictors of 30-day mortality and hospital costs in patients with ventilator-associated pneumonia attributed to potentially antibiotic-resistant gram-negative bacteria. *Chest*.

[18] Kuti E. L., Patel A. A., Coleman C. I. (2008). Impact of inappropriate antibiotic therapy on mortality in patients with ventilator-associated pneumonia and blood stream infection: a meta-analysis. *Journal of Critical Care*.

[19] Chastre J., Wolff M., Fagon J.-Y. (2003). Comparison of 8 vs 15 days of antibiotic therapy for ventilator-associated pneumonia in adults: a randomized trial. *Journal of the American Medical Association*.

[20] Kollef M. H., Silver P., Murphy D. M., Trovillion E. (1995). The effect of late-onset ventilator-associated pneumonia in determining patient mortality. *Chest*.

[21] Canadian Critical Care Trials Group (2006). A randomized trial of diagnostic techniques for ventilator-associated pneumonia. *The New England Journal of Medicine*.

[22] Kumar A., Roberts D., Wood K. E. (2006). Duration of hypotension before initiation of effective antimicrobial therapy is the critical determinant of survival in human septic shock. *Critical Care Medicine*.

[23] Iregui M., Ward S., Sherman G., Fraser V. J., Kollef M. H. (2002). Clinical importance of delays in the initiation of appropriate antibiotic treatment for ventilator-associated pneumonia. *Chest*.

[24] Kett D. H., Cano E., Quartin A. A. (2011). Implementation of guidelines for management of possible multidrug-resistant pneumonia in intensive care: an observational, multicentre cohort study. *The Lancet Infectious Diseases*.

[25] Garnacho-Montero J., Sa-Borges M., Sole-Violan J. (2007). Optimal management therapy for *Pseudomonas aeruginosa* ventilator-associated pneumonia: an observational, multicenter study comparing monotherapy with combination antibiotic therapy. *Critical Care Medicine*.

[26] Sakaguchi M., Shime N., Iguchi N., Kobayashi A., Takada K., Morrow L. E. (2013). Effects of adherence to ventilator-associated pneumonia treatment guidelines on clinical outcomes. *Journal of Infection and Chemotherapy*.

[27] Chastre J., Fagon J.-Y. (2002). Ventilator-associated pneumonia. *American Journal of Respiratory and Critical Care Medicine*.

